# Improving the prioritization of children at the emergency department: Updating the Manchester Triage System using vital signs

**DOI:** 10.1371/journal.pone.0246324

**Published:** 2021-02-09

**Authors:** Joany M. Zachariasse, Ian K. Maconochie, Ruud G. Nijman, Susanne Greber-Platzer, Frank J. Smit, Daan Nieboer, Johan van der Lei, Claudio F. Alves, Henriëtte A. Moll

**Affiliations:** 1 Department of General Paediatrics, Erasmus MC- Sophia Children’s Hospital, University Medical Centre, Rotterdam, The Netherlands; 2 Department of Pediatric Emergency Medicine, Imperial College NHS Healthcare Trust, London, United Kingdom; 3 Section of Paediatric Infectious Diseases, Department of Infectious Diseases, Faculty of Medicine, Imperial College London, London, United Kingdom; 4 Department of Pediatrics and Adolescent Medicine, Medical University Vienna, Vienna, Austria; 5 Department of Pediatrics, Maasstad Hospital, Rotterdam, The Netherlands; 6 Department of Public Health, Erasmus MC-University Medical Center Rotterdam, Rotterdam, The Netherlands; 7 Department of Medical Informatics, Erasmus MC-University Medical Center Rotterdam, Rotterdam, The Netherlands; 8 Department of Pediatrics, Hospital Prof. Dr. Fernando da Fonseca, Lisbon, Portugal; Radboud University Medical Center, NETHERLANDS

## Abstract

**Background:**

Vital signs are used in emergency care settings in the first assessment of children to identify those that need immediate attention. We aimed to develop and validate vital sign based Manchester Triage System (MTS) discriminators to improve triage of children at the emergency department.

**Methods and findings:**

The TrIAGE project is a prospective observational study based on electronic health record data from five European EDs (Netherlands (n = 2), United Kingdom, Austria, and Portugal). In the current study, we included 117,438 consecutive children <16 years presenting to the ED during the study period (2012–2015). We derived new discriminators based on heart rate, respiratory rate, and/or capillary refill time for specific subgroups of MTS flowcharts. Moreover, we determined the optimal cut-off value for each vital sign. The main outcome measure was a previously developed 3-category reference standard (high, intermediate, low urgency) for the required urgency of care, based on mortality at the ED, immediate lifesaving interventions, disposition and resource use. We determined six new discriminators for children <1 year and ≥1 year: “Very abnormal respiratory rate”, “Abnormal heart rate”, and “Abnormal respiratory rate”, with optimal cut-offs, and specific subgroups of flowcharts. Application of the modified MTS reclassified 744 patients (2.5%). Sensitivity increased from 0.66 (95%CI 0.60–0.72) to 0.71 (0.66–0.75) for high urgency patients and from 0.67 (0.54–0.76) to 0.70 (0.58–0.80) for high and intermediate urgency patients. Specificity decreased from 0.90 (0.86–0.93) to 0.89 (0.85–0.92) for high and 0.66 (0.52–0.78) to 0.63 (0.50–0.75) for high and intermediate urgency patients. These differences were statistically significant. Overall performance improved (R^2^ 0.199 versus 0.204).

**Conclusions:**

Six new discriminators based on vital signs lead to a small but relevant increase in performance and should be implemented in the MTS.

## Introduction

Triage is a quick assessment to prioritize patients upon presentation to the emergency department (ED), according to the acuity of their presenting condition. In Europe, the Manchester Triage System (MTS) is the most widely used emergency medical triage system for the triage of adults and children [1]. Previous research has shown that validity of the MTS is moderate to good, with lowest performance in children and elderly [2, 3]. In a recent large prospective study in three European hospitals, sensitivity of the MTS in children ranged from 0.65 (95%CI 0.61–0.70) to 0.83 (95%CI 0.79–0.87), and specificity from 0.83 (95%CI 0.82–0.83) to 0.89 (95%CI 0.88–0.90) [2]. Improvement of the MTS and particularly it’s sensitivity is needed to improve the correct identification of seriously ill children and avoid harm by delays in care [4].

Physiological parameters have been shown early markers of patient deterioration in hospital wards [5–7]. Moreover, children with severe undertriage often have abnormal vital signs [4]. Certain vital parameters, such as oxygen saturation and temperature are integrated within the flowcharts of the MTS, and severe deviations such as airway compromise and shock are included in a descriptive manner. The MTS, however, does not require routine measurement of heart rate, respiratory rate, and capillary refill time, although these vital signs are considered important predictors of severe disease [8–12]. A previous study evaluated the addition of heart rate and respiratory rate to the MTS, but concluded that the use of vital signs did not improve MTS performance [13]. This study, however, added vital signs to all flowcharts in the MTS, applied pre-defined cut-offs, and used hospitalization as the reference standard. We hypothesize that with a different study design and better reference standard, an improvement in performance may be achieved.

The current study aims to develop and validate modifications to the MTS based on vital signs to improve the triage of children at the ED. This study explores the added value of vital signs to specific flowcharts, with optimal cut-off values, using a 3-category reference standard that is a proxy for true patient urgency.

## Methods

The current study was embedded in the TrIAGE project, a European prospective observational study, based on electronic health record data. The study was approved by the participating institutions’ medical ethical committees: Medical Ethics Committee Erasmus MC (MEC-2013-567), Maasstad Ziekenhuis Board of Directors (Protocol L2013-103), Imperial College London Joint Research Compliance Office (Reference number: 14SM2164; Ethics reference number 14/WA/1051), Comissão de Ética para a Saúde do Hospital Prof. Dr. Fernando Fonseca EPE (Reunião de 06 de Dezembro de 2017), Ethik Kommission Medizinische der Medizinischen Unversität Wien (EK Nr: 1405/2014). All waived the requirement for informed consent. We followed the TRIPOD (Transparent Reporting of a Multivariable Prediction Model for Individual Prognosis or Diagnosis) statement for reporting (S6 File) [14].

### Settings, study population and data collection

The TriAGE project is described in detail elsewhere [15]. In short, a cohort was established, consisting of all consecutive ED-visits of children under the age of 16 years. Participating study sites included five EDs in the Netherlands (n = 2), United Kingdom, Austria and Portugal. Enrolment took place during a study period of 8 to 36 months between 2012 and 2015. Nurses routinely recorded patient characteristics, triage details, vital signs and patient disposition in each hospitals’ electronic health record system. These data were automatically extracted, harmonized and checked for quality. The TrIAGE study was based on a convenience sample from five diverse ED settings. Based on projections from the participating hospitals and a pilot in the Erasmus MC, the study was designed to include at least 100 high urgency patients per hospital and at least 100 high urgency patients in the ten most commonly used MTS flowcharts.

### Manchester Triage System

**Fig 1:**
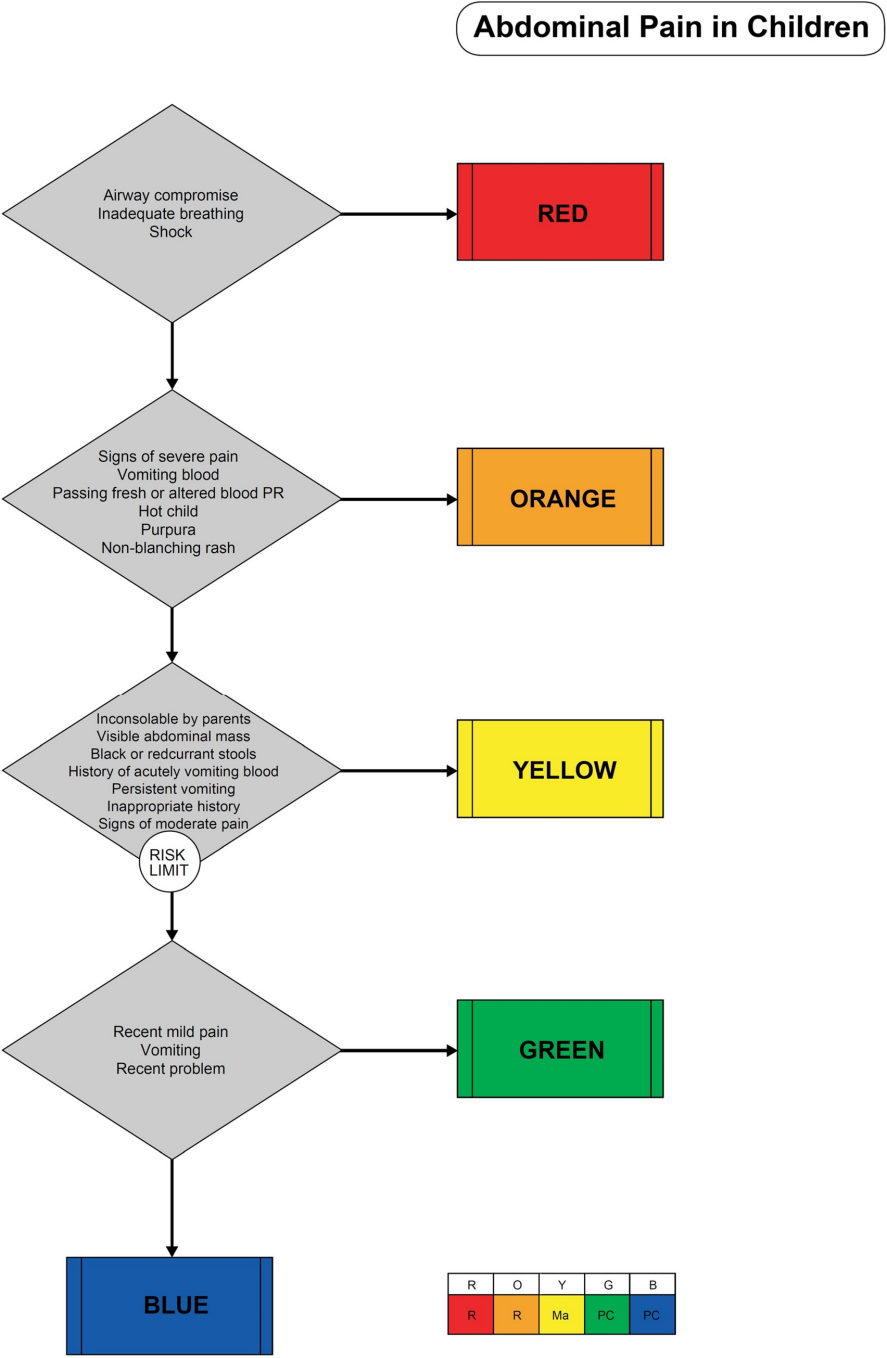
Example MTS flowchart.

The MTS is a flowchart-based emergency medical triage system. It consists of 52 flowcharts that cover almost all presenting problems in the ED. Flowcharts in turn consist of additional signs and symptoms named discriminators that discriminate between five clinical priorities (Immediate, Very urgent, Urgent, Standard or Non-urgent) (Fig 1). Each urgency level has been given a maximum waiting time before first contact with the treating clinician, ranging from 0 minutes (Immediate) to 240 minutes maximum waiting time (Non-urgent) Because of the low proportion of patients in the Immediate (0.8%) and the Non-urgent category (1.4%), we combined the categories Immediate and Very urgent in a high urgency category, and the categories Standard and Non-urgent in a low urgency category for the analysis. For the current study, we excluded all patients with missing MTS urgency or MTS flowchart.

### Vital signs

To improve the MTS, we assessed the value of the vital signs heart rate, respiratory rate and capillary refill time. Other physiological measurements were already included in the MTS (consciousness, temperature, oxygen saturation and increased work of breathing) or not routinely measured at the participating EDs (blood pressure). Vital signs were measured according to each ED’s local practice. Heart rate was measured using a monitor device and respiratory rate was measured manually. Capillary refill time was measured by either pressing the sternum (central CRT) or fingertip (peripheral CRT) for 5 seconds, and >2 seconds was defined as abnormal [8]. We imputed missing values 25 times using a multiple imputation model including predictors, outcome and relevant case-mix variables (S1 File) [16]. The analyses were performed 25 times and pooled for a final result.

### Reference standard

A predefined, composite reference standard was developed for assigning patients to a high, intermediate or low urgency category and serves as a proxy for each child’s true urgency (S1 Table) [15]. Items are based on information from the entire ED visit, including resource use, immediate lifesaving interventions, disposition, and mortality at the ED. These items were selected because they are markers of patient urgency upon presentation to the ED and reflect the time a patient can be allowed to wait before first contact with a physician.

### Principles for improving the MTS

To modify the MTS, one or more vital signs with a specific cut-off should be added as separate discriminator to one or more of the flowcharts. An example could be to add “Heart rate ≥120 beats per minute” as Very urgent discriminator to the flowcharts *Major Trauma* and *Wounds*. This would place all patients with a heart rate ≥120, initially triaged to one of the low urgency categories, in the Very urgent category. According to the MTS’ original principles, discriminators may appear in multiple flowchart but must always lead to the same priority. Discriminators with different levels of severity, lead to different urgency levels. E.g., the MTS discriminator “Very low SaO2” (a saturation <95% on O2 therapy or <90% on air) leads to priority Very urgent, while “Low SaO2” (a saturation <95% on air) leads to priority Urgent throughout the MTS. Adding new MTS discriminators should carefully balance the safety and efficiency of any potential modification. While the goal of triage systems is to recognize patients with the highest clinical priority, it is almost as important to identify the patients with less urgent conditions. In case too many patients are falsely given a high priority, so-called “overtriage”, this group may delay the diagnosis and management of the truly high-urgent patients.

### Statistical analysis

**Fig 2:**
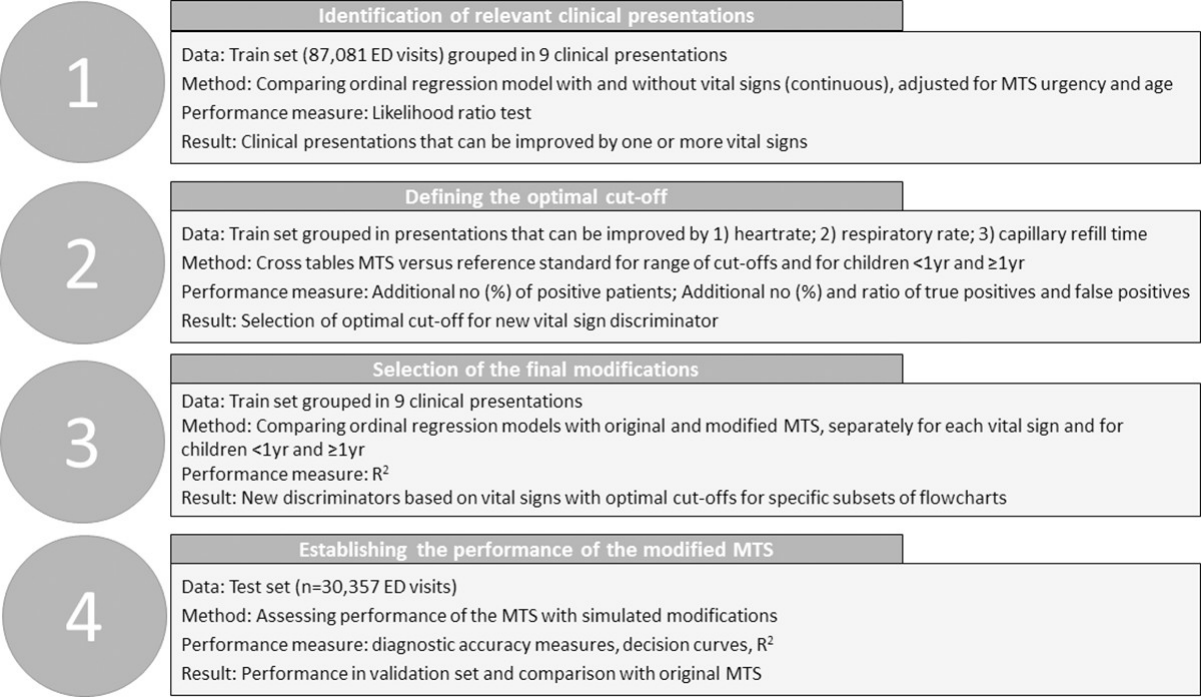
Schematic overview of the methodological approach.

Before conducting the analysis, the dataset was split into a training and a test set, based on time. The initial 75% of arrival dates per setting were assigned to the training set and were used to derive and assess the potential modifications of the MTS. The last 25% of visits were assigned to the test set and used to determine the performance of the modified MTS. To statistically and systematically assess the benefit of adding vital signs as a discriminator, and to take into account the aforementioned principled we approached the analysis in four steps (Fig 2). Details on the methodology can be found in the S2 File.

#### Step 1: Identification of subgroups of MTS flowcharts where a novel vital sign discriminator could have the potential to improve triage

We grouped all MTS flowcharts into nine clinical presentations: Cardiac, Dermatological, Ear Nose Throat, Gastrointestinal, Neurologic or Psychiatric, Respiratory, Trauma or Muscular, General malaise, Uro- or gynaecological and Other, according to a previous study [2]. For each clinical presentation we evaluated an ordinal regression model including MTS urgency level, age (<1 year and ≥1 year), heart rate, respiratory rate and capillary refill time as predictors and the 3-category reference standard as the outcome. Heart rate and respiratory rate were maintained as continuous variables in the analysis. This model assesses the association between vital signs and our outcome measures, adjusted for the already given MTS classification. We applied the likelihood ratio test and considered a p-value of <0.05 statistically significant. As a result, three partly overlapping groups were identified: subgroups of MTS flowcharts that could potentially be improved with the addition of a heart rate-discriminator, flowcharts that could potentially be improved with a respiratory rate discriminator and flowcharts that could potentially be improved with a capillary refill time discriminator.

#### Step 2: Determination of each vital sign’s optimal cut-off to develop definitions for the new vital sign discriminators

For the continuous vital signs heart rate and respiratory rate, a cut-off for both high and intermediate urgency was needed. To determine the optimal cut-off value, we calculated cross tabulations showing the association between the dichotomized MTS (high vs low triage classification) and the dichotomised reference standard (high vs low urgency). We simulated multiple triage modification where a vital sign above a certain cut-off value would place patients in the high urgency triage level. This process was repeated for the range of relevant cut-off values, with increasing steps of 10 beats per minute for heat rate and 5 breaths per minute for respiratory rate. We selected the optimal cut-off value according to three principles, based on consensus from the research team. First, we considered only thresholds with a maximum of 20% increase in the total number of positive patients. Second, we limited the ratio additional true positives: additional false positives, based on consensus from the TrIAGE research group. For the high urgency discriminators, we found a ratio 1 true positive: 15 false positives the maximum acceptable, for the high to intermediate urgency categories this was 1 true positives: 10 false positives. If multiple cut-off values were appropriate, we selected the cut-off with the largest increase in true positive patients.

#### Step 3: Selection of discriminators and subgroups of MTS flowcharts where performance is improved, to determine the final set of modifications

We evaluated ordinal regression models assessing the association between the original MTS and the reference standard and subsequently between the modified MTS and the reference standard. We performed analyses for each of the new discriminators separately. We selected models with a performance that was better than the original MTS as defined by a higher R2.

#### Step 4: Assessment of the modified MTS’ performance as compared with the original MTS

In the test set, we assessed the performance of the modified MTS, i.e. the MTS with the new vital signs discriminators, as compared with the original MTS. We applied the diagnostic accuracy measures sensitivity, specificity and likelihood ratios, constructed decision curves, and calculated Nagelkerke’s R2 in an ordinal analysis. These measures were selected based on our previous study evaluating performance measures in the assessment of modifications for triage systems [17]. Diagnostic accuracy measures were calculated for each of the hospitals individually, and pooled using a random effects model. To determine statistical significance, we used bootstrapping to calculate the differences between sensitivity and specificity of the original and modified MTS in a random sample with replacement and repeated this process 1000 times. We calculated the bootstrapped confidence intervals and p-value. Decision curves provide additional information about clinical value of the proposed modification by incorporating the trade-off between over- and undertriage [18]. Given that most EDs have limited capacity to see all high urgency patients at the same time, and based on consensus from the research group, we do not consider overtriage of more than nine patients acceptable in order to find one true high urgency patient.

To explore, the impact of the multiple imputation on the results, we conducted a sensitivity analysis in the original dataset with missing values. In this analysis, we assumed that the missing vital signs were not considered in the triage decision. All analyses were performed in R version 3.6.1.

## Results

The TrIAGE study is based on a cohort 119,209 ED visits. 1,771 children (1.5%) were excluded due to missing MTS urgency or MTS flowchart leaving a study population of 117,438 children. According to the reference standard 2,964 children (2.5%) were classified as high, 27,826 (24%) as intermediate and 86,648 (74%) as low urgent. The training set consisted of 87,081 children (74%) and the test set of 30,357 (26%) (Table 1). Heart rate was reported in 58% of children, respiratory rate in 48% and capillary refill time in 49% (S1 File).

**Table 1:** Baseline characteristics of the study population.

	Training set (n = 87,081, 74%)	Test set (n = 30,357, 26%)
Age, no. (%)		
< 1 year	13,561 (16)	4,758 (16)
≥ 1 year	73,520 (84)	25,599 (84)
Sex, no. (%)		
Female	39,880 (46)	14,173 (47)
MTS urgency, no. (%)		
Immediate | Very urgent	9,715 (11)	3,747 (12)
Urgent	23,824 (27)	8,138 (27)
Standard | Non-urgent	53,542 (61)	18,472 (61)
Presenting problem, no (%)		
Cardiac	1,010 (1)	388 (1)
Dermatological	11,535 (13)	3,087 (10)
Ear, Nose and Throat	8,215 (9)	3,347 (11)
Gastrointestinal	13,686 (16)	4,531 (15)
Neurologic or psychiatric	3,441 (4)	1,250 (4)
Respiratory	9,640 (11)	4,320 (14)
Trauma or musculoskeletal	16,274 (19)	5,039 (17)
General malaise	7,402 (9)	2,615 (9)
Uro- or gynaecological	1,961 (2)	622 (2)
Other	13,917 (16)	5,158 (17)
Disposition, no. (%)		
ICU or mortality at ED*	467 (0.5)	217 (0.7)
Hospital admission	8,436 (10)	3,004 (10)
Discharge / other	78,178 (89)	27,136 (89)

*Mortality: 12 patients in train set and 4 in test set

### New vital signs discriminators

Heart rate showed a significant association with the reference standard in the cardiac, dermatological, neurologic/psychiatric, and respiratory clinical presentations. For the discriminator very abnormal heart rate (high urgency triage level), we selected as optimal cut-off a heart rate ≥170 beats per minute for children <1 year, and for the discriminator abnormal heartrate (intermediate urgency), ≥160 beats per minute for children <1 year and ≥140 beats per minute for children ≥1 year. A cut-off for children ≥1 year could not be defined, because all of the explored cut-off values led to inacceptable large increases in the number of false positive cases. In the final analyses, only abnormal heartrate was found to improve triage for dermatological and neurologic/psychiatric presentations.

Respiratory rate showed a significant association with the reference standard in the Ear, Nose and Throat (ENT), gastrointestinal, neurologic/psychiatric, respiratory, trauma or muscular, and general malaise clinical presentations. Optimal cut-offs for the discriminator very abnormal respiratory rate (high urgency) were defined at ≥55 breaths per minute for children <1, and ≥45 breaths per minute for children ≥1 year, and ≥45 breaths per minute and ≥35 breaths per minute for the abnormal respiratory rate discriminator (intermediate urgency). All discriminators improved triage in the respiratory and general malaise clinical presentations. The neurologic or psychiatric clinical presentation improved with the abnormal respiratory rate discriminator only.

Capillary refill time showed a significant association with the reference standard in the ENT, gastrointestinal, neurologic/psychiatric, respiratory, and general malaise presentations. Abnormal capillary refill time, applied as an intermediate urgency discriminator, showed the optimal performance, but did not improve triage in any of the presentations in the final analyses and was therefore not selected as discriminator.

Thus, we determined six novel discriminators (Table 2, S3 File). Two discriminators lead to a very urgent classification: “Very abnormal respiratory rate < 1 year”, and “Very abnormal respiratory rate ≥1 year”. Four discriminators lead to an intermediate urgency classification: “Abnormal heart rate <1 year”, “Abnormal heart rate ≥ 1 year”, “Abnormal respiratory rate <1 year”, and “Abnormal respiratory rate ≥ 1 year”. Adding these discriminators to four different clinical presentations, altered 16 MTS flowcharts.

**Table 2:** Definition and application of new vital sign discriminators.

Novel discriminator	Definition	MTS urgency category	Clinical presentation	MTS flowcharts
Very abnormal respiratory rate <1 year	≥ 55 breaths per minute	*Very urgent*	Respiratory General malaise	Asthma, Shortness of breath in children, Unwell child, Irritable child
Very abnormal respiratory rate ≥ 1 year	≥ 45 breaths per minute	*Very urgent*		
Abnormal heart rate <1 year	≥ 160 beats per minute	*Urgent*	Dermatological Neurologic or psychiatric	Rashes, Bites and stings, Burns and scalds, Abscesses and local infections, Wounds, Headache, Fits, Behaving strangely, Overdose and poisoning, Mental illness, Self-harm, Apparently drunk
Abnormal heart rate ≥ 1 year	≥ 140 beats per minute	*Urgent*		
Abnormal respiratory rate <1 year	≥ 45 breaths per minute	*Urgent*	Respiratory General malaise Neurologic or psychiatric	Asthma, Shortness of breath in children, Unwell child, Irritable child, Headache, Fits, Behaving strangely, Overdose and poisoning, Mental illness, Self-harm, Apparently drunk
Abnormal respiratory rate ≥ 1 year	≥ 35 breaths per minute	*Urgent*		

### Performance of the modified MTS

Application of the modified MTS with all new discriminators in our test set reclassified 744 patients (2.5%). The number needed to triage was 41 for one reclassification. Compared with the original MTS, undertriage decreased with 200 patients (0.7%) while an additional 536 patients (1.8%) were overtriaged.

**Fig 3:**
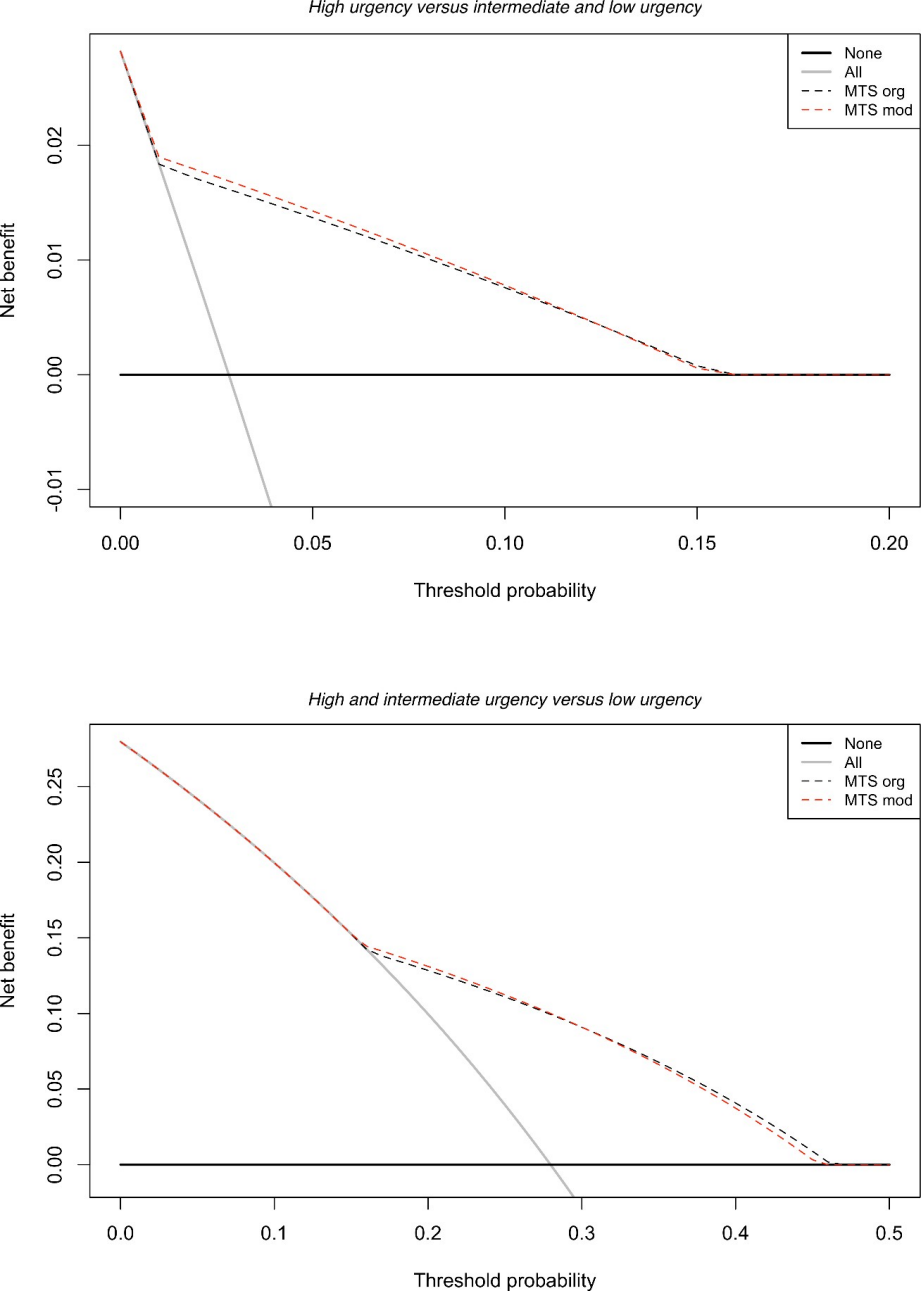
Decision curves comparing the modified MTS with the original.

Sensitivity improved from 0.66 (95%CI 0.60–0.72) to 0.71 (95%CI 0.66–0.75) for the high, and from 0.67 (95%CI 0.54–0.76) to 0.70 (95%CI 0.58–0.80) for the high and intermediate urgency patients. Specificity decreased from 0.90 (95%CI 0.86–0.93) to 0.89 (95%CI 0.85–0.92) for the high, and from 0.66 (95%CI 0.52–0.78) to 0.63 (95%CI 0.50–0.75) for the high and intermediate urgency patients. Both the increase in sensitivity and decrease in specificity were statistically significant, based on the bootstrapped differences between the original and modified MTS (p<0.05, S4 File). According to the decision curves, the modified MTS was the preferred alternative for the range of relevant clinical scenarios (Fig 3). In the ordinal analysis, the modifications improved the R^2^ from 0.199 to 0.204.

In the sensitivity analysis in the dataset without imputation, the number of reclassifications based on the vital signs discriminators was 301 (1.0%). The overall improvement remained, although the effect was slightly smaller (S5 File).

## Discussion

In this European cohort of more than 100,000 paediatric ED visits, we explored the potential to improve the MTS using vital signs. We developed six novel vital signs discriminators that reduced undertriage when each applied to a specific subgroup of MTS flowcharts. The effect on the triage system as a whole was small, but relevant for the individual patient.

The MTS is a widely implemented triage system in Europe. Previously, in a multicentre study of more than 60,000 children in four European hospitals, we proposed modifications to reduce the proportion of overtriage based on the relocation of existing discriminators and demonstrated their safety [13]. Still, sensitivity of the MTS has been moderate, ranging from 0.65 (95%CI 0.61–0.70) to 0.83 (95%CI 0.79–0.87) in a recent prospective study in three European hospitals [2]. Also several single-centre studies on performance of the MTS in children have shown that undertriage still exist and may have important clinical consequences [4, 19].

Physiological measurements have been shown important markers of disease severity in EDs and hospital wards, alone or in combination in early warning scores [7, 10, 20–23]. They are considered crucial in the reliable assessment of any acutely unwell children for the presence of warning signs of underlying serious illness [8]. Moreover, they can be measured routinely by any healthcare professional with experience in the assessment of acutely unwell children. Adding vital signs to a triage system in specific presentations would therefore not greatly affect the ED workflow. Thresholds for abnormal heart rate and respiratory have been proposed [8, 24], but ones that are accurate for and applicable to children presenting with a wide variety of symptoms and optimised for the purpose of triage at the ED remains elusive. A previous study has described the normal ranges and percentiles of vital signs in healthy children [24]. In the ED setting where the majority of children experiences some form of pain, stress or fever, these reference values might be poor predictors of urgency. Clinical reference values such as the APLS guidelines or cut-off values from early warning scores are very heterogeneous and there is no consensus which values should be used in the ED. Moreover, it has been shown that clinical reference values and reference ranges from healthy children are partly overlapping [24]. In a previous large observational study, adding heart rate and respiratory rate to the MTS did not improve its performance [13]. This study, however, added vital signs to all flowcharts in the MTS, and applied pre-defined cut-offs based on previously published 99th percentile values from healthy children [24]. In the current study, we have shown that selecting targeted modifications and using a cut-off value most optimal for the triage setting has the potential to improve the triage of children. The main strength of this study is the large cohort of ED visits from five diverse European EDs. The size of the study population ensured enough power to evaluate modifications in the high urgency population, that only comprises 2% of the study sample. Moreover, we evaluated potential vital signs modifications in a thorough and systematic approach, thereby aiming to identify any relevant discriminators.

However, some limitations have to be acknowledged. In our study vital signs were missing in 42% to 52% of the ED visits. This proportion is in line with previous studies reporting on vital signs measurements in the ED [25–27]. To deal with the missing values we used a multiple imputation approach to reduce bias [16]. Moreover, a sensitivity analysis in the original dataset without imputation showed that results were largely similar. The number of reclassifications caused by our proposed modifications is relatively small. It is possible that by limiting the amount of overtriage we considered acceptable, valuable modifications have been missed and a higher sensitivity could have been achieved. Based on the existing structure of the MTS, we selected only two age-specific cut-off values, one for children <1 year and one for children ≥1 year. Although the use more age-categories would have made the cut-off values more precise, we intended to adhere to the MTS’ original principles, to facilitate implementation in clinical practice. Paediatric Early Warning Scores (PEWS) are scoring systems based on physiological parameters, that combine the number of abnormal measurements, and the amount of deviation from the normal into a single score [10, 15]. To fully use the potential of vital signs, a PEWS can be used as an additional tool in de triage process, although further research is needed to determine its value in different subgroups of patients. Finally, we used split-sample validation to assess performance of the MTS’ modifications and pooled the different performance measures using a random effects model to capture heterogeneity in performance across settings. However, we did not formally externally validate the modified MTS. We propose to evaluate and validate the new modifications after implementation in practice, to gain further understanding of performance in practice in different clinical settings.

The proposed modification results in a decrease of undertriage of 0.7% (n = 200 patients) at the cost of an increase in overtriage of 1.8% (n = 536). Reducing undertriage is important to improve patient safety. As it decreases delays in care it may prevent morbidity or even mortality. Overtriage does not have a direct impact on patient health and thus, a certain amount of overtriage can be considered acceptable. There is, however, a trade-off. When the amount of overtriage is too large, it will affect waiting times for the truly high urgent patients. In addition, many EDs have limited resources and thus require adequate prioritization. Based on the consensus from the TrIAGE research group, we propose that overtriage of not more than nine patients is acceptable in order to find one additional true high urgent patients, although we acknowledge that in some settings this number may be lower. Our current modification results in the overtriage of less than three patients for each correctly identified high urgent patients, which is well within these pre-specified limits. Since our study used an exhaustive approach we believe it demonstrates the maximal improvement that can be achieved using vital signs within the MTS. The proposed modifications are ready for implementation and validation in clinical practice. Moreover, as we developed targeted modifications to the MTS, the alterations do not have a large impact on the nurses’ workload with regard to vital signs’ measurements, increasing acceptability in clinical practice. Future studies should focus on novel markers for urgency in children such as nurse or parental gut feeling. Following triage, patients can be monitored for clinical deterioration using re-triage, PEWS [10], or scoring systems for specific subgroups of children [28, 29].

In conclusion, novel age-specific modifications based on the vital signs heart rate and respiratory rate in specific subgroups of flowcharts improve the performance of the MTS. We propose to include these evidence-based modifications in the MTS.

## Supporting information

S1 Table 3-category reference standard as proxy for true patient urgency. (DOCX)

S1 File Missing vital signs measurements and multiple imputation. (DOCX)

S2 File Methodology. (DOCX)

S3 File Intermediate results step 1 to 3. (DOCX)

S4 File Performance of modified MTS. (DOCX)

S5 File Sensitivity analysis based on missing values. (DOCX)

S6 File TRIPOD checklist. (DOCX)

## Abbreviations

CRT: capillary refill time
ED: emergency department
ENT: Ear Nose and Throat
MTS: Manchester Triage System
PEWS: Paediatric Early Warning Score
