# Supplementary material for: Improving the prioritization of children at the emergency department: Updating the Manchester Triage System using vital signs
Source: PLoS One. 2021 Feb 9;16(2):e0246324. doi: 10.1371/journal.pone.0246324 (PMC7872278; doi:10.1371/journal.pone.0246324)
Supplement: S1 Table — (DOCX) [file pone.0246324.s001.docx]

**S1 Table. 3-category reference standard as proxy for true patient urgency**

| **3-category reference standard** | |
| --- | --- |
| High urgency | - Mortality at the ED, *and/or* - ICU admission immediately after the ED visit, *and/or* - Immediate lifesaving interventions*, *and/or* - Oxygen administration |
| Intermediate urgency | - Hospital admission immediately after the ED visit, *and/or* - IV medication or fluids or inhalation medication at the ED, *and/or* - >1 of the following: Radiology; Lab test; Oral medication |
| Low urgency | - None of the above |

***** Immediate lifesaving interventions are defined as any of the following [1,2] :
 - airway/breathing support (e.g. intubation or emergent noninvasive positive pressure ventilation);
 - electrical therapy (e.g. defibrillation, emergent cardioversion or external pacing);
 - emergency procedures (e.g. chest needle decompression, pericardiocentesis, or open
 thoracotomy)
 - haemodynamic support (e.g. significant IV fluid in case of hypotension, blood
 administration or control of major bleeding) or emergency medications (e.g. atropine,
 adenosine, inotropics, epinephrine, nalaxon, dextrose in case of hypoglycaemia)

**References**

1. Lee JY, Oh SH, Peck EH, et al. The validity of the Canadian Triage and Acuity Scale in predicting resource utilization and the need for immediate life-saving interventions in elderly emergency department patients. Scand J Trauma Resusc Emerg Med 2011;19:68.

2. Platts-Mills TF, Travers D, Biese K, et al. Accuracy of the Emergency Severity Index triage instrument for identifying elder emergency dep
