# Supplementary material for: Improving the prioritization of children at the emergency department: Updating the Manchester Triage System using vital signs
Source: PLoS One. 2021 Feb 9;16(2):e0246324. doi: 10.1371/journal.pone.0246324 (PMC7872278; doi:10.1371/journal.pone.0246324)
Supplement: S1 File — (DOCX) [file pone.0246324.s002.docx]

**S1 File. Missing vital signs measurements and multiple imputation**

Vital signs were missing with variables rates across the hospitals (Table 1). We examined the relationship between missingness of the data and other patient characteristics (Tables 2-4). There was a strong association between whether physiological measurements were missing and type of presenting complaint, triage urgency and disposition after the ED visit. More items were missing in patients presenting with trauma or muscular problems, and in patients of low urgency (as reflected by triage urgency and disposition after the ED visit). This supports our assumption that the data are “missing at random”.

We developed an imputation model that included general patient characteristics, date and time variables, triage items, physiological parameters, and variables related to diagnostics, therapy, and disposition (Table 5).

Missing data were handled by 25-fold multiple imputation using Fully Conditional Specification (FCS) implemented by the MICE package. FCS specifies the multivariate imputation model on a variable-by-variable basis by a set of conditional densities, one for each incomplete variable. Starting from an initial imputation, FCS draws imputations by iterating over the conditional densities. Data were assumed to be missing at random.

**Table 1. Proportion of missing vital signs in the different hospitals**

|  | **Heart rate (n, %)** | **Respiratory rate, n(%)** | **Capillary refill time, n(%)** |
| --- | --- | --- | --- |
| Erasmus MC | 8,464 (47) | 11,819 (66) | 10,934 (61) |
| Maasstad Hospital | 6,378 (61) | 8,710 (83) | 8,022 (77) |
| St. Mary’s Hospital | 3,028 (19) | 3,653 (23) | 5, 823 (37) |
| Hospital Fernando da Fonseca | 19,106 (36) | 19,106 (36) | 17,814 (34) |
| Vienna General Hospital | 12,331 (61) | 17,527 (87) | 17,331 (86) |

**Table 2. Proportion of missing data according to presenting problem**

|  | **Heart rate (n, %)** | **Respiratory rate, n(%)** | **Capillary refill time, n(%)** |
| --- | --- | --- | --- |
| Cardiac | 32 (2·3) | 381 (27) | 455 (33) |
| Dermatological | 8,328 (57) | 8,835 (60) | 8,530 (58) |
| Ear, Nose and Throat | 4,959 (43) | 5,378 (47) | 5,217 (45) |
| Gastrointestinal | 7,272 (40) | 8,888 (49) | 8,359 (46) |
| Neurologic or Psychiatric | 1,097 (23) | 2,065 (44) | 2,148 (46) |
| Respiratory | 864 (6.2) | 4,099 (29) | 4,796 (34) |
| Trauma or muscular | 16,008 (75) | 17,157 (81) | 16,516 (77) |
| General malaise | 1,905 (19) | 3,487 (35) | 3,696 (37) |
| Uro- or gynaecological | 1,266 (49) | 1,414 (55) | 1,319 (51) |
| Other | 7,576 (40) | 9,111 (48) | 8,888 (47) |

**Table 3. Proportion of missing data according to triage urgency**

|  | **Heart rate (n, %)** | **Respiratory rate, n(%)** | **Capillary refill time, n(%)** |
| --- | --- | --- | --- |
| Emergent or very urgent | 2,418 (18) | 4,076 (30) | 4,334 (32) |
| Urgent | 14,144 (44) | 18,442 (58) | 17,357 (54) |
| Standard/non-urgent | 32,745 (45) | 38,297 (53) | 38,233 (53) |

**Table 4. Proportion of missing data according to disposition after ED discharge**

|  | **Heart rate (n, %)** | **Respiratory rate, n(%)** | **Capillary refill time, n(%)** |
| --- | --- | --- | --- |
| ICU admission | 98 (14) | 230 (34) | 337 (49) |
| Hospital admission | 3,124 (27) | 5,439 (48) | 5,254 (46) |
| Discharge / other | 46,085 (44) | 55,146 (52) | 54,333 (52) |

**Table 5 Imputation model**

| **Type of information** | **Variables** |
| --- | --- |
| General patient characteristics | Hospital (cat); Age (cont.); Gender (M/F); Referral type (Self / GP / Emergency services / Other) |
| Date and time | Arrival month (1-12); Arrival hour (morning/evening/night); Arrival day (week/weekend) |
| Triage items | MTS flowchart (cat); MTS urgency (1-5); MTS painscore (cont |
| Physiological parameters | Heart rate (cont); Respiratory rate (cont);  Temperature (cont); Systolic bloodpressure (cont); Diastolic bloodpressure (cont); Saturation (cont); Capillary refill (dich); Consciousness (normal / decreased / unconsciousness); |
| Diagnostics, Therapy, Disposition | Any lab (Y/N); Any imaging (Y/N); Any cultures (Y/N); Oxygen therapy (Y/N); Oral meds (Y/N); Inhalation meds (Y/N); IV meds or fluids (Y/N); Immediate lifesaving interventions (Y/N); Disposition (Mortality or ICU admission/ Admission / Other (cat) |
