# Supplementary material for: Improving the prioritization of children at the emergency department: Updating the Manchester Triage System using vital signs
Source: PLoS One. 2021 Feb 9;16(2):e0246324. doi: 10.1371/journal.pone.0246324 (PMC7872278; doi:10.1371/journal.pone.0246324)
Supplement: S2 File — (DOCX) [file pone.0246324.s003.docx]

**S2 File. Methodology**

The analysis of our study was approached in 4 steps detailed below.

**Step 1. Identification of relevant clinical presentations**

We grouped all MTS flowcharts into nine clinical presentations: Cardiac, Dermatological, Ear Nose Throat, Gastrointestinal, Neurologic or Psychiatric, Respiratory, Trauma or Muscular, General malaise, Uro- or gynaecological and Other, according to a previous study (Table 1).[1] We choose to combine flowcharts in clinical presentations because this represents the clinical practice of triage where often multiple related flowcharts can be used to adequately triage a patient. Moreover, combining related flowcharts increases the number of patients in the relatively small high urgency category and could improve the power to detect improvement of our modifications.

For each clinical presentation we evaluated an ordinal regression model including MTS urgency level, age (<1 year and ≥1 year), heart rate, respiratory rate and capillary refill time as predictors and the 3-category reference standard as the outcome. Heart rate and respiratory rate were maintained as continuous variables in the analysis. This model assesses the association between vital signs and our outcome measures, adjusted for the already given MTS classification. We applied the likelihood ratio test and considered a p-value of <0.05 statistically significant.

If none of the vital signs in a model was significantly associated with the outcome, it was supposed that this clinical presentation could not be improved by adding vital signs. If one, two or all vital signs were significant with a p<0.05 these vital signs were selected for further investigation in relation to the specific clinical presentation.

As a result, three partly overlapping groups were identified: presentations that could potentially be improved with the addition of a heart rate-discriminator, presentations that could potentially be improved with a respiratory rate discriminator and presentations that could potentially be improved with a capillary refill time discriminator.

**Table 1.** Grouping of flowcharts into clinical presentations

| **Clinical presentation** | **MTS Flowcharts** |
| --- | --- |
| **Cardiac** | Chest pain, Palpitations |
| **Dermatological** | Rashes, Bites and stings, Burns and scalds, Abscesses and local infections, Wounds |
| **Ear, Nose and Throat** | Sore throat, Facial problems, Ear problems |
| **Gastrointestinal** | Abdominal pain in adults, Abdominal pain in children, Diarrhoea and vomiting, GI bleeding |
| **Neurologic or Psychiatric** | Headache, Fits, Collapsed adult, Behaving strangely, Overdose and poisoning, Mental illness, Self-harm, Apparently drunk |
| **Respiratory** | Asthma, Shortness of breath in adults, Shortness of breath in children |
| **Trauma or muscular** | Limb problems, Major trauma, Neck pain, Back pain, Torso injury, Falls, Assault, Head injury, Limping child |
| **General malaise** | Unwell adult, Unwell child, Irritable child |
| **Uro- or gynaecological** | Urinary problems, Testicular pain, PV bleeding, Sexually acquired infection, Pregnancy |
| **Other** | Major incidents primary, Worried parent, Dental problems, Exposure to chemicals, Foreign body, Diabetes, Eye problems, Allergy, Crying baby, General/Other |

**Step 2. Defining the optimal cut-off**

The vital signs heart rate and respiratory rate were previously maintained continuous but required a cut-off to define which values were considered abnormal. For these vital signs, a cut-off for both high and intermediate urgency was needed. Capillary refill time was available as a dichotomous variable (normal or abnormal). For this vital sign, the analysis should determine whether a positive discriminator should lead to a high and intermediate urgency.

In the analysis, we combined all relevant clinical presentations for each vital sign. Thus, for the analysis of heart rate, we combined all presentations where heart rate had the potential to improve triage according to the analysis in step 1. This was needed because in all different presentations the cut-off value of a vital sign should be the same according to the principles of the MTS.

We analysed the cut-off for heart rate and respiratory rate separately in children <1 year and ≥1 year, thereby aiming to take into account the age-related normal ranges of the vital signs. Moreover, this is consistent with the fever discriminator currently used in the MTS that gives a different priority to children <1 year and ≥1 year.

For a potential modification leading to a high urgency classification, we dichotomised both the MTS and reference standard in a high urgency versus an intermediate and low urgency group. For a potential modification leading to an intermediate urgency classification, we dichotomised the MTS and reference standard in a high and intermediate urgency versus a low urgency group.

We started each analysis with a cross table assessing the association between the dichotomized MTS and the dichotomised reference standard. We calculated the number of total positive patients, true positive patients, and false positive patients. Next, we simulated a modification where a vital sign above a certain cut-off value would place patients in the high urgency level. This process was repeated for the range of relevant cut-off values, with increasing steps of 10 beats per minute for heat rate and 5 breaths per minute for respiratory rate. For each simulated modification we calculated the increase in the total number of positive patients. In addition, we calculated the additional true positive patients and the additional false positive patients and the ratio true positive : false positive patients. We selected the optimal cut-off value according to three principles, based on consensus from the research team. First, we considered only thresholds with a maximum of 20% increase in the total number of positive patients. This, because the number of patients that can be seen immediately in the high urgency categories is limited, and a too large increase in this category makes the triage system inefficient. Second, we limited the ratio additional true positives : additional false positives. For the high urgency discriminators, we found a ratio 1 true positive : 15 false positives the maximum acceptable, for the high to intermediate urgency categories this was 1 true positives : 10 false positives. If multiple cut-off values were appropriate, we selected the cut-off with the largest increase in true positive patients.

**Step 3. Selection of the final modifications**

In the third step, we simulated that the novel discriminators were added to the MTS, to determine in which clinical presentations the triage was improved. To evaluate performance, in each of the clinical presentation we evaluated an ordinal regression model assessing the association between the original MTS and the reference standard and subsequently between the modified MTS and the reference standard. We performed analyses for each of the new discriminators separately. We selected models with a performance that was better than the original MTS as defined by a higher R^2^. We choose the R^2^ based on its statistical properties and because it is one of the few performance measures applicable to ordinal models. [2,3] Ultimately, this selection resulted in a final set of proposed modifications: novel vital-sign discriminators and a subgroup of clinical presentations where triage could be improved.

**Step 4. Establishing the performance of the modified MTS**

Finally, we assessed the performance of the modified MTS, i.e. the MTS with the new vital signs discriminators, as compared with the original MTS. We applied diagnostic accuracy measures and constructed a decision curve based on a dichotomous analysis, and calculated Nagelkerke’s R^2^ in an ordinal analysis. These measures were selected based on our previous study evaluating performance measures in the assessment of modifications for triage systems.[4] We constructed 2x2 contingency tables for the original and modified MTS versus the reference standard, and calculated sensitivity, specificity, positive and negative likelihood ratios. For the performance of the high urgency discriminators, we dichotomized the MTS into the high urgency category versus the combination of the intermediate and low urgency categories. For the performance of the intermediate urgency categories, we dichotomized the MTS into the combination of the high and intermediate urgency categories versus the low urgency category. The reference standard was dichotomized in the same manner. The results were calculated for each of the hospitals individually, and pooled using a random effects model. To determine statistical significance, we used bootstrapping to calculate the differences between sensitivity and specificity of the original and modified MTS in a random sample with replacement and repeated this process 1000 times. We calculated the bootstrapped confidence intervals and p-value.

In addition, we constructed decision curves comparing the original and modified MTS. Decision curves provide additional information about clinical value of the proposed modification by incorporating the trade-off between over- and undertriage.[5,6] In short, the Y-axis represents the benefit of each approach, expressed as net benefit. The highest value indicates the preferred alternative. The X-axis represents the range of preferences one might have regarding not missing any high urgent patients versus the wish to avoid a large increase in overtriage. This is needed because each proposed modification that will correctly assign some ill patients to a high urgency level will also unintentionally result in some additional low urgency patients falsely triaged to a high urgency level. Although health workers and health care settings may differ as to how much under- and overtriage is acceptable, there is only a range of such values applicable to the clinical practice in emergency departments. We propose that, given the risks involved when missing a high urgency patient, it would be considered unreasonable to accept a more than 20% risk of high urgency to wait before being seen by a physician in any setting. For high and intermediate urgency patients, we propose this risk is at most 40%. Of course, in some settings the acceptable risk might be lower. On the other hand, many emergency departments face large volumes of patients and have only limited capacity to see all high urgency patients at the same time. Therefore, we do not consider overtriage of more than nine patients acceptable in order to find one true high urgency patient. Therefore, we consider the relevant x-axis range between 0.1 and 0.2 for the high urgency patients and between 0.1 and 0.3 for the intermediate urgency patients, and the approach with the highest benefit within this range the preferred alternative.

Finally, we calculated Nagelkerke’s R^2^ as a marker of overall performance. The calculation was based on an ordinal regression model with MTS urgency (in 3 categories) as the independent variable and the reference standard as the outcome.

**References**

1. Zachariasse JM, Seiger N, Rood PP, et al. Validity of the Manchester Triage System in emergency care: A prospective observational study. *PLoS One* 2017;12(2):e0170811.

2. Zachariasse JM, van der Hagen V, Seiger N, et al. Performance of triage systems in emergency care: a systematic review and meta-analysis. *BMJ Open* 2019;9(5):e026471.

3. Steyerberg EW, Vickers AJ, Cook NR, et al. Assessing the performance of prediction models: a framework for traditional and novel measures. *Epidemiology* 2010;21(1):128-38.

4. Zachariasse JM, Nieboer D, Oostenbrink R, et al. Multiple performance measures are needed to evaluate triage systems in the emergency department. *J Clin Epidemiol* 2018;94:27-34.

5. Vickers AJ, Elkin EB. Decision curve analysis: a novel method for evaluating prediction models. *Med Decis Making* 2006;26(6):565-74.

6. Vickers AJ, van Calster B, Steyerberg EW. A simple, step-by-step guide to interpreting decision curve analysis. *Diagn Progn Res* 2019;3:18.
