# Supplementary material for: Improving the prioritization of children at the emergency department: Updating the Manchester Triage System using vital signs
Source: PLoS One. 2021 Feb 9;16(2):e0246324. doi: 10.1371/journal.pone.0246324 (PMC7872278; doi:10.1371/journal.pone.0246324)
Supplement: S3 File — (DOCX) [file pone.0246324.s004.docx]

**S3 File. Intermediate results step 1 to 3**

**Step 1. Identification of relevant clinical presentations**

Based on the likelihood ratio test (p<0.05), eight of the nine clinical presentations could be improved by adding one or more vital signs. Heart rate had the potential to improve the cardiac, dermatological, Ear Nose and Throat (ENT), neurologic or psychiatric, and respiratory presentations. Respiratory rate could potentially improve the ENT, gastrointestinal, neurologic or psychiatric, respiratory, trauma or muscular, and general malaise presentations. Capillary refill time could potentially improve the ENT, gastrointestinal, neurologic or psychiatric, respiratory, and general malaise presentations. The triage of uro- or gynaecological presentations could not be improved by any of the vital signs (Table 1).

**Table 1.** Results of the likelihood ratio test, assessing the relation between heart rate, respiratory rate and capillary refill time, adjusted for age and triage urgency level, with the 3-category reference standard

|  | **Total, n** | **Chi-square statistic (p-value)** | | |
| --- | --- | --- | --- | --- |
|  |  | **HR** | **RR** | **CRT** |
| **Cardiac** | 1010 | 18.56  (<0.0001)* | 0.06  (0.8023) | 1.55  (0.2139) |
| **Dermatological** | 11,535 | 6.97  (0.0083) * | 1.84  (0.1753) | 3.05  (0.0806) |
| **Ear, Nose and Throat** | 8,215 | 31.26  (<0.0001) * | 19.30  (<0.0001) * | 5.98  (0.0145) * |
| **Gastrointestinal** | 13,686 | 0.35  (0.5537) | 4.56  (0.0327) * | 17.03  (<0.0001) * |
| **Neurologic or psychiatric** | 3,441 | 12.85  (0.0003) * | 5.10  (0.0240) * | 14.17  (0.0002) * |
| **Respiratory** | 9,640 | 12.31  (0.0005) * | 167.20  (<0.0001) * | 9.64  (0.0019) * |
| **Trauma or muscular** | 16,274 | 1.59  (0.2070) | 22.60  (<0.0001) * | 2.19  (0.1388) |
| **General malaise** | 7,402 | 0.15  (0.7004) | 56.65  (<0.0001) * | 34.10  (<0.0001) * |
| **Uro- or gynaecological** | 1,961 | 0.12  (0.7294) | 2.47  (0.1163) | 0.87  (0.3510) |

MTS= Manchester Triage System; HR= Heart rate; RR= Respiratory Rate; CRT= Capillary Refill Time
*p<0.05

**Step 2. Defining the optimal cut-off**

We assessed a range of heart rate and respiratory rates and both cut-offs for capillary refill time. (Tables 2-10) Based on the pre-specified increase in the number of positive patients, and the ratio true positive : false positive, we determined for each of the vital signs the optimal cut-off (Table 11).

For the potential discriminator “Very abnormal heart rate” it was not possible to define a cut-off because any increase in true positives would lead to an inacceptable high number of false positives. Therefore, we had to exclude this potential discriminator from further analyses.

**Table 2.** Selecting the cut-off for the high urgency heart rate discriminator “Very abnormal heart rate < 1 year”

|  | nP, n | dP, n(%) | nTP, n | nFP, n | dTP, n | dFP, n | Ratio |
| --- | --- | --- | --- | --- | --- | --- | --- |
| **MTS original** | **1663** | **-** | **289** | **1374** | **-** | **-** | **-** |
| HR ≥160 | 2050 | 387 (23) | 314 | 1737 | 24 | 363 | 1:15 |
| HR ≥170 * | 1839 | 176 (11) | 304 | 1535 | 15 | 161 | 1:11 |
| HR ≥180 | 1733 | 70 (4) | 298 | 1435 | 9 | 61 | 1:7 |
| HR ≥190 | 1683 | 20 (1) | 291 | 1392 | 2 | 19 | 1:12 |
| HR ≥200 | 1666 | 3 (0) | 289 | 1377 | 0 | 3 | - |

nP= Absolute number of positive patients; dP= Additional number of positive patients compared with original MTS; nTP= Absolute number of True Positives; nFP= Absolute number of False Positives; dTP= Additional number of true positives compared with original MTS; dFP= Additional number of false positives compared with original MTS; Ratio= Ratio dTP : dFP; MTS= Manchester Triage System; HR= Heart rate
*= Final cut-off selected

**Table 3.** Selecting the cut-off for the intermediate urgency heart rate discriminator “Abnormal heart rate < 1 year”

|  | nP, n | dP, n(%) | nTP, n | nFP, n | dTP, n | dFP, n | Ratio |
| --- | --- | --- | --- | --- | --- | --- | --- |
| **MTS original** | **2154** | **-** | **1323** | **831** | **-** | **-** | **-** |
| HR ≥150 | 2707 | 553 (26) | 1468 | 1239 | 145 | 408 | 1:3 |
| HR ≥160 * | 2441 | 287 (13) | 1407 | 1035 | 84 | 204 | 1:2 |
| HR ≥170 | 2282 | 128 (6) | 1365 | 917 | 42 | 86 | 1:2 |
| HR ≥180 | 2201 | 47 (2) | 1337 | 864 | 14 | 33 | 1:2 |
| HR ≥190 | 2165 | 11 (1) | 1326 | 839 | 3 | 8 | 1:3 |
| HR ≥200 | 2156 | 2 (0) | 1323 | 832 | 0 | 1 | 1:3 |

nP= Absolute number of positive patients; dP= Additional number of positive patients compared with original MTS; nTP= Absolute number of True Positives; nFP= Absolute number of False Positives; dTP= Additional number of true positives compared with original MTS; dFP= Additional number of false positives compared with original MTS; Ratio= Ratio dTP : dFP; MTS= Manchester Triage System; HR= Heart rate
*= Final cut-off selected

**Table 4.** Selecting the cut-off for the high urgency heart rate discriminator “Very abnormal heart rate ≥ 1 year”

|  | nP, n | dP, n(%) | nTP, n | nFP, n | dTP, n | dFP, n | Ratio |
| --- | --- | --- | --- | --- | --- | --- | --- |
| **MTS original** | **5027** | **-** | **852** | **4175** | **-** | **-** | **-** |
| HR ≥140 | 7620 | 2593 (52) | 922 | 6699 | 70 | 2524 | 1:36 |
| HR ≥150 | 6382 | 1355 (27) | 891 | 5491 | 39 | 1316 | 1:34 |
| HR ≥160 | 5724 | 697 (14) | 877 | 4848 | 25 | 673 | 1:27 |
| HR ≥170 | 5322 | 295 (6) | 865 | 4458 | 13 | 283 | 1:22 |
| HR ≥180 | 5163 | 136 (3) | 860 | 4303 | 8 | 128 | 1:16 |
| HR ≥190 | 5079 | 52 (1) | 855 | 4224 | 3 | 49 | 1:16 |
| HR ≥200 | 5040 | 13 (0) | 853 | 4187 | 1 | 12 | 1:19 |

nP= Absolute number of positive patients; dP= Additional number of positive patients compared with original MTS; nTP= Absolute number of True Positives; nFP= Absolute number of False Positives; dTP= Additional number of true positives compared with original MTS; dFP= Additional number of false positives compared with original MTS; Ratio= Ratio dTP : dFP; MTS= Manchester Triage System; HR= Heart rate
*= Final cut-off selected

**Table 5.** Selecting the cut-off for the intermediate urgency heart rate discriminator “Abnormal heart rate ≥ 1 year”

|  | nP, n | dP, n(%) | nTP, n | nFP, n | dTP, n | dFP, n | Ratio |
| --- | --- | --- | --- | --- | --- | --- | --- |
| **MTS original** | **11,634** |  | **5618** | **6016** |  |  |  |
| HR ≥140 * | 13195 | 1561 (13) | 5930 | 7265 | 312 | 1249 | 1:4 |
| HR ≥150 | 12401 | 767 (7) | 5787 | 6613 | 170 | 597 | 1:4 |
| HR ≥160 | 11992 | 358 (3) | 5705 | 6286 | 88 | 270 | 1:3 |
| HR ≥170 | 11776 | 142 (1) | 5656 | 6120 | 39 | 104 | 1:3 |
| HR ≥180 | 11690 | 56 (0) | 5633 | 6057 | 15 | 41 | 1:3 |
| HR ≥190 | 11649 | 15 (0) | 5622 | 6027 | 4 | 11 | 1:2 |
| HR ≥200 | 11637 | 3 (0) | 5619 | 6019 | 1 | 3 | 1:3 |

nP= Absolute number of positive patients; dP= Additional number of positive patients compared with original MTS; nTP= Absolute number of True Positives; nFP= Absolute number of False Positives; dTP= Additional number of true positives compared with original MTS; dFP= Additional number of false positives compared with original MTS; Ratio= Ratio dTP : dFP; MTS= Manchester Triage System; HR= Heart rate
*= Final cut-off selected

**Table 6.** Selecting the cut-off for the high urgency respiratory rate discriminator “Very abnormal respiratory rate < 1 year”

|  | nP, n | dP, n(%) | nTP, n | nFP, n | dTP, n | dFP, n | Ratio |
| --- | --- | --- | --- | --- | --- | --- | --- |
| **MTS original** | **2375** | **-** | **353** | **2022** | **-** | **-** | **-** |
| RR ≥ 45 | 3316 | 941 (40) | 403 | 2913 | 51 | 890 | 1:18 |
| RR ≥ 50 | 2925 | 550 (23) | 394 | 2531 | 42 | 508 | 1:12 |
| RR ≥ 55 * | 2702 | 327 (14) | 377 | 2324 | 25 | 302 | 1:12 |
| RR ≥ 60 | 2600 | 225 (9) | 371 | 2229 | 19 | 207 | 1:11 |
| RR ≥ 65 | 2430 | 55 (2) | 361 | 2069 | 8 | 47 | 1:6 |
| RR ≥ 70 | 2403 | 28 (1) | 357 | 2046 | 5 | 24 | 1:5 |

nP= Absolute number of positive patients; dP= Additional number of positive patients compared with original MTS; nTP= Absolute number of True Positives; nFP= Absolute number of False Positives; dTP= Additional number of true positives compared with original MTS; dFP= Additional number of false positives compared with original MTS; Ratio= Ratio dTP : dFP; MTS= Manchester Triage System; RR= Respiratory rate
*= Final cut-off selected

**Table 7.** Selecting the cut-off for the intermediate urgency respiratory rate discriminator “Abnormal respiratory rate < 1 year”

|  | nP, n | dP, n(%) | nTP, n | nFP, n | dTP, n | dFP, n | Ratio |
| --- | --- | --- | --- | --- | --- | --- | --- |
| **MTS original** | **4037** | **-** | **2258** | **1779** | **-** | **-** | **-** |
| RR ≥ 45 * | 4626 | 589 (15) | 2469 | 2157 | 211 | 378 | 1:2 |
| RR ≥ 50 | 4369 | 332 (8) | 2385 | 1983 | 128 | 204 | 1:2 |
| RR ≥ 55 | 4224 | 187 (5) | 2330 | 1893 | 73 | 114 | 1:2 |
| RR ≥ 60 | 4163 | 126 (3) | 2312 | 1851 | 54 | 72 | 1:1 |
| RR ≥ 65 | 4063 | 26 (1) | 2272 | 1791 | 14 | 12 | 1:1 |
| RR ≥ 70 | 4051 | 14 (0) | 2267 | 1784 | 9 | 5 | 2:1 |

nP= Absolute number of positive patients; dP= Additional number of positive patients compared with original MTS; nTP= Absolute number of True Positives; nFP= Absolute number of False Positives; dTP= Additional number of true positives compared with original MTS; dFP= Additional number of false positives compared with original MTS; Ratio= Ratio dTP : dFP; MTS= Manchester Triage System; RR= Respiratory rate
*= Final cut-off selected

**Table 8.** Selecting the cut-off for the high urgency respiratory rate discriminator “Very abnormal respiratory rate ≥ 1 year”

|  | nP, n | dP, n(%) | nTP, n | nFP, n | dTP, n | dFP, n | Ratio |
| --- | --- | --- | --- | --- | --- | --- | --- |
| **MTS original** | **5897** | **-** | **948** | **4949** | **-** | **-** | **-** |
| RR ≥ 30 | 14372 | 8475 (144) | 1145 | 13227 | 197 | 8278 | 1:42 |
| RR ≥ 35 | 9700 | 3803 (64) | 1081 | 8619 | 133 | 3670 | 1:27 |
| RR ≥ 40 | 8032 | 2135 (36) | 1040 | 6992 | 92 | 2043 | 1:22 |
| RR ≥ 45 * | 6750 | 853 (14) | 1002 | 5748 | 54 | 799 | 1:15 |
| RR ≥ 50 | 6357 | 460 (8) | 979 | 5378 | 31 | 429 | 1:14 |
| RR ≥ 55 | 6159 | 262 (4) | 968 | 5190 | 21 | 241 | 1:12 |
| RR ≥ 60 | 6081 | 184 (3) | 963 | 5118 | 16 | 169 | 1:11 |
| RR ≥ 65 | 5930 | 33 | 950 | 4980 | 3 | 31 | 1:12 |
| RR ≥ 70 | 5912 | 15 | 948 | 4964 | 1 | 14 | 1:17 |

nP= Absolute number of positive patients; dP= Additional number of positive patients compared with original MTS; nTP= Absolute number of True Positives; nFP= Absolute number of False Positives; dTP= Additional number of true positives compared with original MTS; dFP= Additional number of false positives compared with original MTS; Ratio= Ratio dTP : dFP; MTS= Manchester Triage System; RR= Respiratory rate
*= Final cut-off selected

**Table 9.** Selecting the cut-off for the intermediate urgency respiratory rate discriminator “Abnormal respiratory rate ≥1 year”

|  | nP, n | dP, n(%) | nTP, n | nFP, n | dTP, n | dFP, n | Ratio |
| --- | --- | --- | --- | --- | --- | --- | --- |
| **MTS original** | **21915** | **-** | **10543** | **11372** | **-** | **-** | **-** |
| RR ≥ 30 | 26564 | 4649 (21) | 11495 | 15069 | 952 | 3697 | 1:4 |
| RR ≥ 35 * | 23808 | 1893 (9) | 11030 | 12778 | 487 | 1405 | 1:3 |
| RR ≥ 40 | 22907 | 992 (5) | 10840 | 12066 | 298 | 694 | 1:2 |
| RR ≥ 45 | 22285 | 370 (2) | 10674 | 11611 | 131 | 239 | 1:2 |
| RR ≥ 50 | 22104 | 189 (1) | 10612 | 11492 | 69 | 120 | 1:2 |
| RR ≥ 55 | 22017 | 102 (0) | 10582 | 11435 | 39 | 63 | 1:2 |
| RR ≥ 60 | 21987 | 72 (0) | 10570 | 11417 | 27 | 44 | 1:2 |
| RR ≥ 65 | 21925 | 10 (0) | 10547 | 11378 | 4 | 6 | 1:2 |
| RR ≥ 70 | 21919 | 4 (0) | 10544 | 11375 | 2 | 3 | 1:2 |

nTP= Absolute number of True Positives; nFP= Absolute number of False Positives; Se= Sensitivity; Sp= nP= Absolute number of positive patients; dP= Additional number of positive patients compared with original MTS; nTP= Absolute number of True Positives; nFP= Absolute number of False Positives; dTP= Additional number of true positives compared with original MTS; dFP= Additional number of false positives compared with original MTS; Ratio= Ratio dTP : dFP; MTS= Manchester Triage System; RR= Respiratory rate
*= Final cut-off selected

**Table 10.** Selecting the cut-off for the discriminator “Abnormal capillary refill time”

|  | nP, n | dP, n(%) | nTP, n | nFP, n | dTP, n | dFP, n | Ratio |
| --- | --- | --- | --- | --- | --- | --- | --- |
| **MTS original** | **7438** | **-** | **1255** | **6183** | **-** | **-** | **-** |
| Cut-off 1  (“High urgent”) | 7929 | 491 (7) | 1271 | 6658 | 16 | 475 | 1:30 |
| **MTS original** | 19452 | - | 10572 | 8880 | - | - | - |
| Cut-off 2 (“Urgent”) * | 19633 | 181 (1) | 10627 | 9005 | 55 | 126 | 1:2 |

nTP= Absolute number of True Positives; nFP= Absolute number of False Positives; Se= Sensitivity; Sp= nP= Absolute number of positive patients; dP= Additional number of positive patients compared with original MTS; nTP= Absolute number of True Positives; nFP= Absolute number of False Positives; dTP= Additional number of true positives compared with original MTS; dFP= Additional number of false positives compared with original MTS; Ratio= Ratio dTP : dFP; MTS= Manchester Triage System; RR= Respiratory rate
*= Final cut-off selected

**Table 11.** Definitions of new vital sign discriminators

| **Discriminator** | **Urgency category** | **Potential clinical presentations** | **Definition** | |
| --- | --- | --- | --- | --- |
|  |  |  | <1 year | >= 1 year |
| Very abnormal  heart rate | *Very urgent* | Cardiac Dermatological ENT Neurologic or psychiatric  Respiratory | ≥170 | NA * |
| Abnormal  heart rate | *Urgent* |  | ≥160 | ≥140 |
| Very abnormal  respiratory rate | *Very urgent* | ENT  Gastrointestinal Neurologic or psychiatric Respiratory  Trauma or muscular General malaise | ≥55 | ≥45 |
| Abnormal  respiratory rate | *Urgent* |  | ≥45 | ≥35 |
| Abnormal  capillary refill time | *Urgent* | ENT  Gastrointestinal  Neurologic or psychiatric  Respiratory  General malaise | ≥2 seconds ** | |

* Not possible to define an acceptable cut-off; ** Cut-off pre-specified prior to analysis

**Step 3. Selection of the final modifications**

We compared the performance of the modified MTS including the new vital signs discriminators with the original MTS separately for each clinical presentation and each vital sign (Table 12). Abnormal heart rate improved triage in the dermatological and neurologic or psychiatric presentations. Respiratory rate improved triage in the respiratory, general malaise, and neurologic or psychiatric presentations. Capillary refill time did not improve performance according to the R^2^ in any clinical presentation.

**Table 12.** Comparison of performance original MTS and modified MTS with one of the vital sign discriminators added, based on R^2^

|  | **Original MTS** | **Very abnormal HR (<1 year)** | **Abnormal  HR** | **Very abnormal RR** | **Abnormal  RR** | **Abnormal  CRT** |
| --- | --- | --- | --- | --- | --- | --- |
| Cardiac | 0.100 | 0.100 | 0.100 | - | - | - |
| Dermatological | 0.095 | 0.094 | 0.097 * | - | - | - |
| Ear, Nose and Throat | 0.010 | 0.010 | 0.010 | 0.010 | 0.009 | 0.010 |
| Gastrointestinal | 0.127 | - | - | 0.125 | 0.123 | 0.126 |
| Neurologic or psychiatric | 0.189 | 0.188 | 0.194 * | 0.185 | 0.192 * | 0.181 |
| Respiratory | 0.232 | 0.230 | 0.227 | 0.241 * | 0.238 * | 0.230 |
| Trauma or muscular | 0.145 | - | - | 0.144 | 0.144 |  |
| General malaise | 0.130 | - | - | 0.133 * | 0.137 * | 0.122 |

HR= Heart rate; RR= Respiratory rate; CRT= Capillary Refill Time; MTS= Manchester Triage System
* Improvement in R^2^
