# Supplementary material for: Improving the prioritization of children at the emergency department: Updating the Manchester Triage System using vital signs
Source: PLoS One. 2021 Feb 9;16(2):e0246324. doi: 10.1371/journal.pone.0246324 (PMC7872278; doi:10.1371/journal.pone.0246324)
Supplement: S4 File — (DOCX) [file pone.0246324.s005.docx]

**S4 File. Performance of modified MTS**

**Table 1.** Reclassification table of original versus modified MTS in the test set

|  | **Modified MTS, n** | | | | |
| --- | --- | --- | --- | --- | --- |
| **Original MTS, n** |  | **Emergent / Very urgent** | **Urgent** | **Standard / Non Urgent** | **Total** |
|  | **Emergent/ very urgent** | 3,747 | 0 | 0 | 3,747 |
|  | **Urgent** | 111 | 8,027 | 0 | 8,138 |
|  | **Standard / Non Urgent** | 109 | 524 | 17,839 | 18,472 |
|  | **Total** | 3,967 | 8,551 | 17,839 | 30,357 |

**Table 2.**  Original MTS versus reference standard classification

|  | **Reference standard, n** | | | | |
| --- | --- | --- | --- | --- | --- |
| **Original MTS, n** |  | **High urgency** | **Intermediate urgency** | **Low urgency** | **Total** |
|  | **Emergent/ very urgent** | 582 | 2052 | 1113 | 3747 |
|  | **Urgent** | 161 | 2702 | 5276 | 8138 |
|  | **Standard / Non Urgent** | 112 | 2881 | 15479 | 18472 |
|  | **Total** | 855 | 7634 | 21867 | 30357 |

**Table 3.**  Modified MTS versus reference standard classification

|  | **Reference standard, n** | | | | |
| --- | --- | --- | --- | --- | --- |
| **Modified MTS, n** |  | **High urgency** | **Intermediate urgency** | **Low urgency** | **Total** |
|  | **Emergent/ very urgent** | 610 | 2147 | 1210 | 3967 |
|  | **Urgent** | 152 | 2779 | 5620 | 8551 |
|  | **Standard / Non Urgent** | 93 | 2709 | 15037 | 17839 |
|  | **Total** | 855 | 7634 | 21867 | 30357 |

**Table 4.**  Performance of modified MTS according to diagnostic accuracy measures

|  | **Sensitivity** | **Specificity** | **Positive likelihood ratio** | **Negative likelihood ratio** |
| --- | --- | --- | --- | --- |
| High urgency versus intermediate and low urgency | | | | |
| Original MTS | 0.66 (0.60-0.72) | 0.90 (0.86-0.93) | 6.5 (4.9-8.2) | 0.37 (0.32-0.43) |
| Modified MTS | 0.71 (0.66-0.75) | 0.89 (0.85-0.92) | 6.3 (4.7-8.0) | 0.33 (0.28-0.38) |
| High and intermediate urgency versus low urgency | | | | |
| Original MTS | 0.67 (0.54-0.76) | 0.66 (0.52-0.78) | 2.0 (1.5-2.4) | 0.50 (0.42-0.59) |
| Modified MTS | 0.70 (0.58-0.80) | 0.63 (0.50-0.75) | 1.9 (1.5-2.3) | 0.48 (0.40-0.56) |

**Table 5.** Bootstrapped differences between the original and modified MTS

|  | **Pooled mean difference** | **95% confidence interval** | **p-value** |
| --- | --- | --- | --- |
| Sensitivity | 0.040 | 0.085 to 0.071 | p<0.05 |
| Specificity | -0.010 | -0.015 to -0.005 | p<0.05 |

**Fig 1.**  Decision curves comparing the modified MTS with the original

**
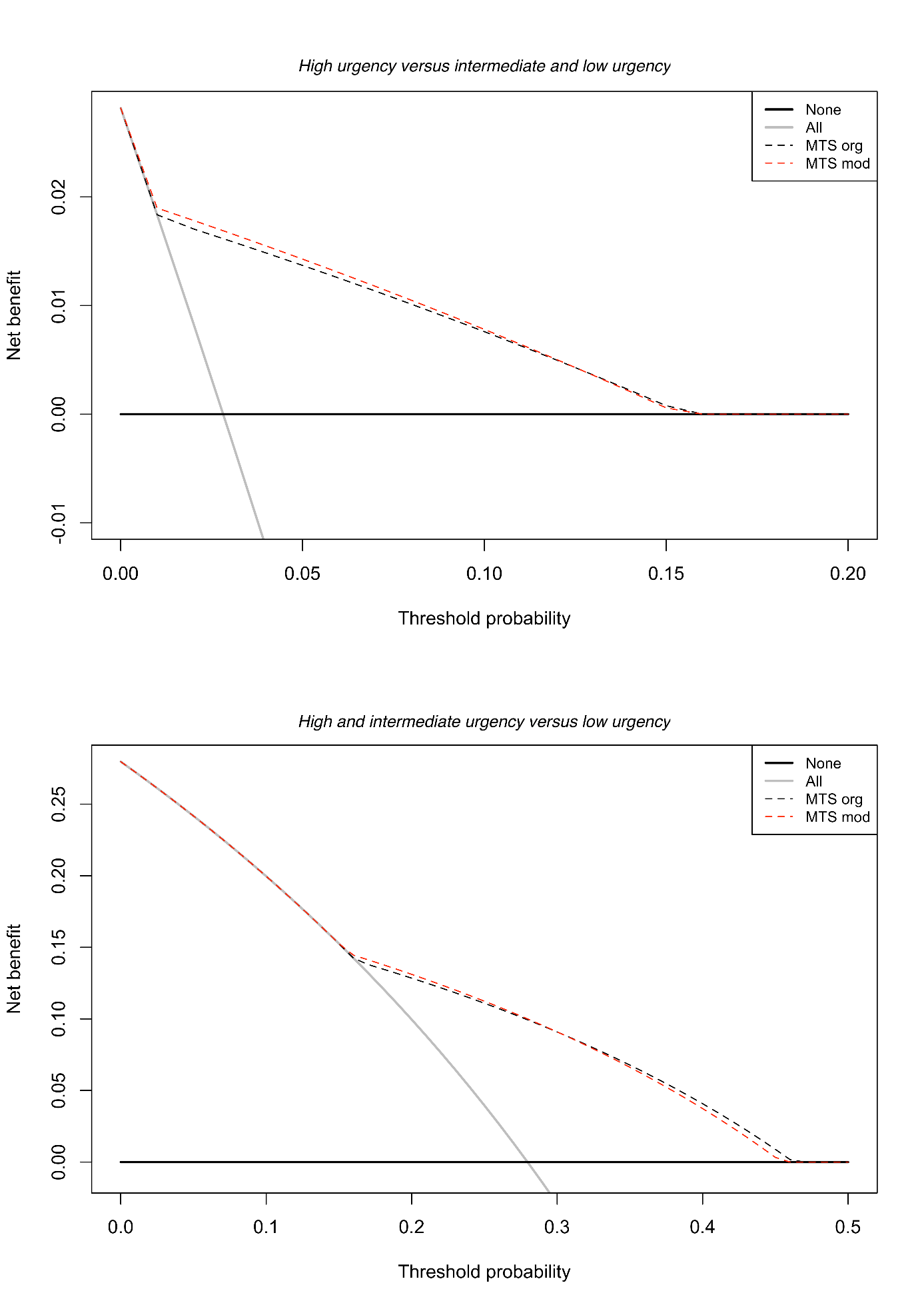
**
