# Supplementary material for: Improving the prioritization of children at the emergency department: Updating the Manchester Triage System using vital signs
Source: PLoS One. 2021 Feb 9;16(2):e0246324. doi: 10.1371/journal.pone.0246324 (PMC7872278; doi:10.1371/journal.pone.0246324)

**S5 File. Sensitivity analysis based on missing values**

In the sensitivity analysis, missing vital signs remained missing and were not considered in the triage decision.

**Table 1.** Reclassification table of original versus modified MTS in the sensitivity analysis

|  | **Modified MTS, n** | | | | |
| --- | --- | --- | --- | --- | --- |
| **Original MTS, n** |  | **Emergent / Very urgent** | **Urgent** | **Standard / Non Urgent** | **Total** |
|  | **Emergent/ very urgent** | 3,747 | 0 | 0 | 3,747 |
|  | **Urgent** | 55 | 8,083 | 0 | 8,138 |
|  | **Standard / Non Urgent** | 49 | 197 | 18,226 | 18,472 |
|  | **Total** | 3,851 | 8,280 | 18,226 | 30,357 |

**Table 2.**  Original MTS versus reference standard classification in the sensitivity analysis

|  | **Reference standard, n** | | | | |
| --- | --- | --- | --- | --- | --- |
| **Original MTS, n** |  | **High urgency** | **Intermediate urgency** | **Low urgency** | **Total** |
|  | **Emergent/ very urgent** | 582 | 2,052 | 1113 | 3,747 |
|  | **Urgent** | 161 | 2,702 | 5,276 | 8,138 |
|  | **Standard / Non Urgent** | 112 | 2,881 | 15,479 | 18,472 |
|  | **Total** | 855 | 7,634 | 21,867 | 30,357 |

**Table 3.**  Modified MTS versus reference standard classification in the sensitivity analysis

|  | **Reference standard, n** | | | | |
| --- | --- | --- | --- | --- | --- |
| **Modified MTS, n** |  | **High urgency** | **Intermediate urgency** | **Low urgency** | **Total** |
|  | **Emergent/ very urgent** | 602 | 2,097 | 1,152 | 3,851 |
|  | **Urgent** | 154 | 2,739 | 5,387 | 8,280 |
|  | **Standard / Non Urgent** | 99 | 2,798 | 15,328 | 18,226 |
|  | **Total** | 855 | 7,634 | 21,867 | 30,357 |

**Table 4.** Performance of the modified MTS compared to the original in the sensitivity analysis

|  | **Sensitivity** | **Specificity** | **Positive likelihood ratio** | **Negative likelihood ratio** | **R^2^** |
| --- | --- | --- | --- | --- | --- |
| High urgency versus intermediate and low urgency | | | | |  |
| Original MTS | 0.66  (0.60-0.72) | 0.90  (0.86-0.93) | 6.5  (4.9-8.2) | 0.37  (0.32-0.43) | - |
| Modified MTS | 0.70  (0.67-0.74) | 0.89 (0.86-0.92) | 6.7  (4.5-8.8) | 0.33 (0.29-0.37) | - |
| High and intermediate urgency versus low urgency | | | | |  |
| Original MTS | 0.67  (0.54-0.78) | 0.66  (0.52-0.78) | 2.0  (1.5-2.4) | 0.50 (0.42-0.59) | - |
| Modified MTS | 0.68 (0.55-0.79) | 0.65 (0.51-0.77) | 2.0 (1.5-2.4) | 0.49 (0.40-0.57) | - |
| Ordinal | | | | |  |
| Original | - | - | - | - | 0.199 |
| Modified MTS | - | - | - | - | 0.204 |

**Fig 1.** Decision curve of the modified MTS compared to the original in the sensitivity analysis


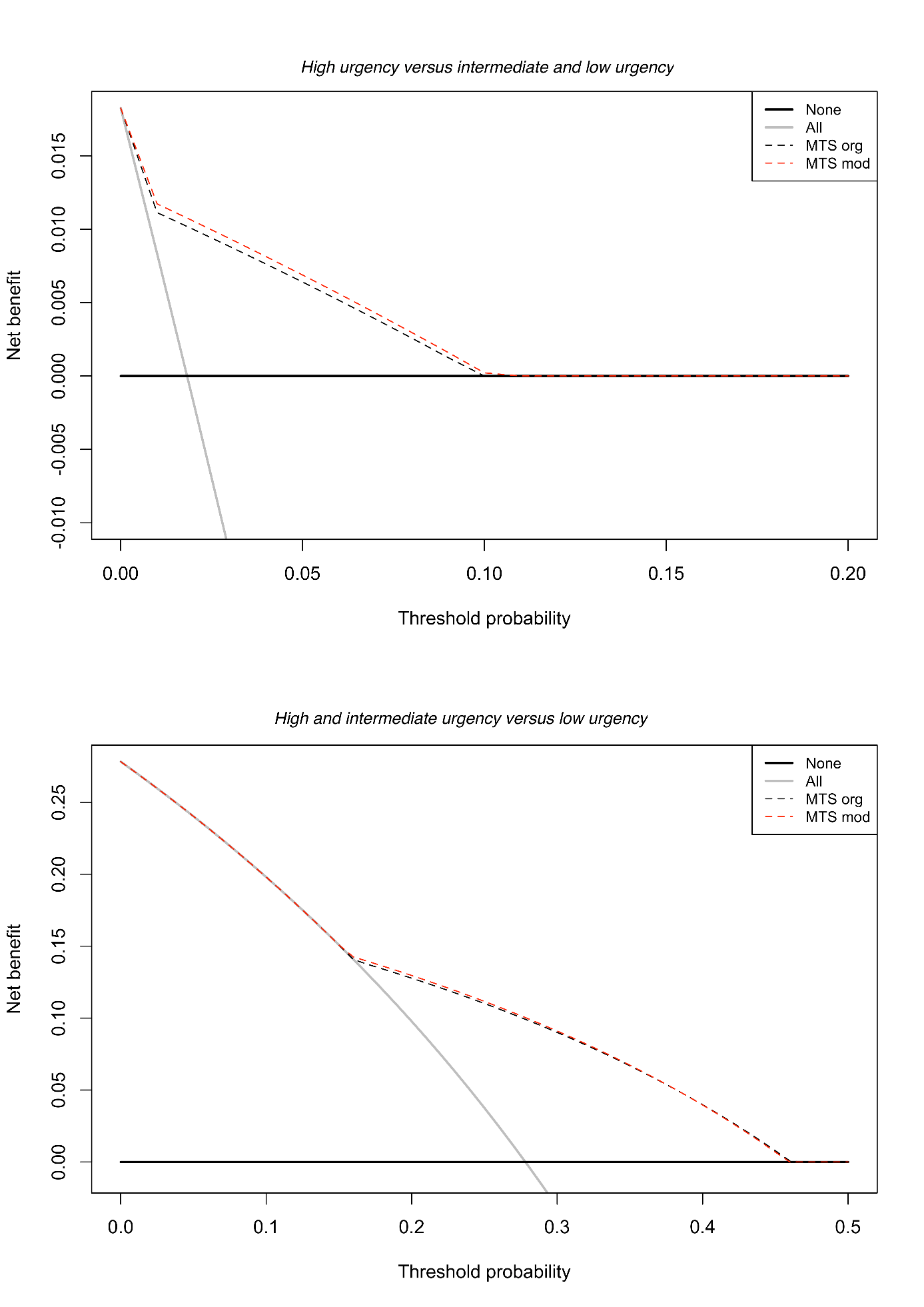

Supplement: S5 File — (DOCX) [file pone.0246324.s006.docx]
